# Apparent diffusion coefficient of normal breast tissue during the menstrual cycle at 3 Tesla

**DOI:** 10.1186/bcr2957

**Published:** 2011-11-04

**Authors:** N AlRashidi, T Ahearn, B Jagpal, T Redpath, F Gilbert

**Affiliations:** 1 University of Aberdeen, UK

## Introduction

Diffusion-weighted magnetic resonance imaging (DW-MRI) is a quantitative MRI technique that provides physiological information by measuring the degree of water molecule diffusion within the extracellular space. It gives a quantitative measurement known as the apparent diffusion coefficient (ADC) value. The aim of the study is to show the influence of the menstrual cycle on breast ADC values and the relationship of the ADC to transverse relaxation (T2) value.

## Methods

Female volunteers had one MRI scan per week over 4 weeks using a 3 T MRI scanner. The ADC of the fibroglandular tissue was measured using a single-shot SE-EPI with four *b *values (0, 50, 150, and 800 s/mm^2^). The T2 relaxation time was measured using T2w turbo spin echo (TSE) with four echo times (20, 40, 60, and 80 ms). ADC and T2 maps were generated automatically by standard Philips software.

## Results

The study was performed on 11 healthy volunteers (23 to 41 years old) with a regular menstrual cycle. There is no significant difference between ADC and T2 values for the 4 weeks. Pearson's correlation coefficient indicated a negative correlation between ADC and T2 values. See Table 1.

**Table 1:** 

Parameter	Week 1	Week 2	Week 3	Week 4
ADC (×10^-3 ^mm^2^/s)				
Fibroglandular tissue	1.7 ± 0.2	1.7 ± 0.3	1.7 ± 0.3	1.7 ± 0.3
T2 (ms)				
Fibroglandular tissue	57.6 ± 7	58 ± 7	58.7 ± 8	60 ± 10
Adipose tissue	78 ± 4	77 ± 4	77 ± 4	77 ± 4

Data presented as mean ± SD.

## Conclusion

The study shows that ADC values are not affected by the normal hormonal fluctuations during the menstrual cycle.

